# An improved distance measure between the expression profiles linking co-expression and co-regulation in mouse

**DOI:** 10.1186/1471-2105-7-44

**Published:** 2006-01-26

**Authors:** Ryung S Kim, Hongkai Ji, Wing H Wong

**Affiliations:** 1 Department of Neurology, Harvard Medical School, Boston, MA 02115, USA; 2 Department of Statistics, Harvard University, Cambridge, MA 02138, USA; 3 Department of Statistics, Stanford University, Stanford, CA 94305, USA; 4 Department of Medical Oncology, Dana-Farber Cancer Institute, Boston, MA02115, USA

## Abstract

**Background:**

Many statistical algorithms combine microarray expression data and genome sequence data to identify transcription factor binding motifs in the low eukaryotic genomes. Finding cis-regulatory elements in higher eukaryote genomes, however, remains a challenge, as searching in the promoter regions of genes with similar expression patterns often fails. The difficulty is partially attributable to the poor performance of the similarity measures for comparing expression profiles. The widely accepted measures are inadequate for distinguishing genes transcribed from distinct regulatory mechanisms in the complicated genomes of higher eukaryotes.

**Results:**

By defining the regulatory similarity between a gene pair as the number of common known transcription factor binding motifs in the promoter regions, we compared the performance of several expression distance measures on seven mouse expression data sets. We propose a new distance measure that accounts for both the linear trends and fold-changes of expression across the samples.

**Conclusion:**

The study reveals that the proposed distance measure for comparing expression profiles enables us to identify genes with large number of common regulatory elements because it reflects the inherent regulatory information better than widely accepted distance measures such as the Pearson's correlation or cosine correlation with or without log transformation.

## Background

Many statistical algorithms combine microarray expression data and genome sequence data to find transcription factor binding motifs (TFBMs) in the low eukaryotic genomes. An early work searches for the regulatory motifs that are associated with significant mean expression changes when they are in the promoter regions of genes; the motifs are then clustered according to their contributions across the arrays [1]. Several approaches fit expression data to motif occurrences by multivariate linear regression model; thereafter, the motifs are selected by classical covariate selection procedures [2-4]. These works were validated in Saccharomyces cerevisiae genome. Finding cis-regulatory elements in higher eukaryotes, however, remains a challenge. In higher eukaryotes, the gene expression clusters often do not lead to successful identification of the transcription factor binding sites. The difficulty arises from two aspects.

First, the complex regulatory mechanisms of higher eukaryotes impede the search of the genomes. Transcription factor binding sites, usually short (6–12 bases), may appear in far upstream, e.g., 20,000 bases upstream from the transcription starting site, in the introns and even in the downstream regions. Furthermore, the transcription factors work in combinations [5-8]. Several approaches are proposed to overcome the difficulty. For example, studies showed that cross-species genome alignment could guide the search for functional regulatory elements [9-12]. Only about 5% of the mammalian genome is under purifying selection [13], and we can study a small subset of genome that is more likely to have important functions by focusing on common non-coding regions across the species.

Second, the widely accepted distance measures for comparing expression profiles are inadequate for distinguishing genes from distinct regulatory mechanisms in higher eukaryotes. The quality of the distance measure is fundamental for high-level analysis methods such as clustering algorithms to identify co-expressed gene groups. From our experience in microarray data analysis, often genes with similar expression patterns share little common TFBMs in their promoter regions. We will propose a distance measure for expression profiles that correlates better with the regulatory distance than the widely used distance measures such as one minus correlation and one minus cosine correlation. Several studies have improved the quality of distance measures by accounting for the technical noise during the hybridization of mRNA on gene chips [14]; they, however, do not account for the regulatory information [14].

## Results

### Data description

The performances of different distance measures were compared on seven experiments that consist overall 288 mouse oligonucleotide microarrays. The regulatory pathways involved in the experiments shall vary across the data sets. Su et al. [15] generated expression data, 90 arrays, from dissected mouse samples across 45 tissue types: the data represent a substantial description of the normal murine transcriptome because the samples mainly come from the normal physiological state. Storch et al. [16] generated circadian gene expressions, 24 arrays, from mouse liver and heart: mice were synchronized to a 12-h light/dark cycle for 2 weeks and to the dim light for 42-h before the tissues were collected at 4-h intervals over two circadian cycles. Wang et al. [17] generated gene expressions, 35 arrays, during the preimplantation development over 12 time points from germinal vesicle stage oocyte to expanded blastocyst. Zhao et al. [18] generated muscle regeneration genes expressions, 54 arrays, across 27 time points up to 40 days after injecting a toxin into the mouse gastrocnemius muscle. We have neocortex developmental gene expressions, 17 arrays, across the developmental time courses from embryonic 8.5 days to 10 days postnatal. In addition, we used 2 mouse expression profile data sets from the Public Expression Profiling Resource [19]: 1) Forty samples from a BALB/CJ murine model of human asthma that used the ragweed pollen to sensitize and challenge the mice, 2) thirty brain hippocampus samples from neurofibromin-1 heterozygous and control mice, 15 samples each, collected from 10 to 32 days postnatal. The mRNA samples from Su et al. [15] were hybridized on Affymetrix MG_U74A chips. The samples from all other data sets were hybridized on either MOE430A or MG_U74av2 chips.

We then mapped 147 known mouse TFBM matrices to the regions from 5000 base upstream to 1000 base downstream relative to transcription starting site of all genes in two mouse gene chips. Only top 10% regions most well aligned across the genomes of three species were used in the motif mapping. The known binding motifs and the information on their corresponding binding proteins were obtained from the online database TRANSFAC [20]. These known TFBMs represent a small portion of all transcription factors in mouse.

### Linking co-expression and co-regulation, in mouse

**Figure 1:**
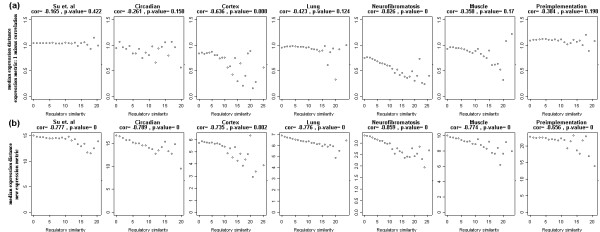
**Correlation of median expression distance with the regulatory similarity in seven data sets**. Each point is the observed median expression distance of gene pairs as a function of the number of common TFBMs in the pairs. Two expression distance measures are used: (a) 1 minus correlation, and (b) the new expression distance measure. For each data set, the correlation between median expression distance and regulatory similarity is computed. To calculate the significance of such correlations, the mapping between genes and their promoter regions were permuted 500 times. When fewer than 5 gene pairs have certain regulatory similarity, the median expression distance is computed after combining nearest regulatory similarities to make each point in the plots represent at least 5 gene pairs. The genes that share large number of common TFBMs are more likely to have correlated expression patterns: sometimes, the effect is present only when they share enough common TFBMs. Table 1 summarizes the results with 7 different distance measures. The figure and the Table 1 show that, while all other distance measures perform similar, the new distance measure correlates best with the regulatory similarity. Only the new distance measure correlates significantly with all seven data sets.

For each of seven data sets, we first selected 1,000 probe sets with the most variable expression patterns. Then for each pair of genes, we identified the common TFBMs in the promoter regions of both genes. The redundancy of some probe sets with a common target gene or of some genes with overlapping promoter regions was taken into account (See Methods). We defined the measure of the regulatory similarity between a gene pair as the number of the common TFBMs in their promoter regions (See Methods). A significantly large number of TFBMs in the promoter region of a gene may indicate the validity of their regulatory role on the gene [21]. Here, we extended the idea to define the regulatory similarity between two genes. Then, we defined the expression distance between a gene pair as 1 minus correlation between two profiles. Figure 1a shows the observed median expression distance of gene pairs as a function of the regulatory similarity between the two genes. The figure demonstrates that genes that share large number of common TFBMs are more likely to have highly correlated expression patterns: sometimes, the effect is present only when they share enough common TFBMs. For each data set, the correlation was computed between the median expression distance and regulatory similarity. To calculate the significance of such correlation, we permuted the mapping between genes and their promoter regions 500 times. Note that some p-values are not statistically significant in the figure. In the next section, we propose a simple distance measure between the expression profiles that correlates stronger with the regulatory similarity.

### A new distance measure for comparing expression profiles

When the medians of two expression profiles over *n *samples (*x*_1_, ..., *x*_n_), (*y*_1_, ..., *y*_n_) are *m*(*x*) and *m*(*y*), we define the distance between two profiles as following:

∑i=1n{log⁡2(xi/m(x))−log⁡2(yi/m(y))}2
 MathType@MTEF@5@5@+=feaafiart1ev1aaatCvAUfKttLearuWrP9MDH5MBPbIqV92AaeXatLxBI9gBaebbnrfifHhDYfgasaacH8akY=wiFfYdH8Gipec8Eeeu0xXdbba9frFj0=OqFfea0dXdd9vqai=hGuQ8kuc9pgc9s8qqaq=dirpe0xb9q8qiLsFr0=vr0=vr0dc8meaabaqaciaacaGaaeqabaqabeGadaaakeaadaGcaaqaamaaqahabaWaaiWaaeaacyGGSbaBcqGGVbWBcqGGNbWzdaWgaaWcbaGaeGOmaidabeaakiabcIcaOmaalyaabaGaemiEaG3aaSbaaSqaaiabdMgaPbqabaaakeaacqWGTbqBcqGGOaakcqWG4baEcqGGPaqkaaGaeiykaKIaeyOeI0IagiiBaWMaei4Ba8Maei4zaC2aaSbaaSqaaiabikdaYaqabaGccqGGOaakdaWcgaqaaiabdMha5naaBaaaleaacqWGPbqAaeqaaaGcbaGaemyBa0MaeiikaGIaemyEaKNaeiykaKcaaiabcMcaPaGaay5Eaiaaw2haamaaCaaaleqabaGaeGOmaidaaaqaaiabdMgaPjabg2da9iabigdaXaqaaiabd6gaUbqdcqGHris5aaWcbeaaaaa@5541@

The distance measure is equivalent as the Euclidian distance between two standardized profiles (x˜1,⋯,x˜n
 MathType@MTEF@5@5@+=feaafiart1ev1aaatCvAUfKttLearuWrP9MDH5MBPbIqV92AaeXatLxBI9gBaebbnrfifHhDYfgasaacH8akY=wiFfYdH8Gipec8Eeeu0xXdbba9frFj0=OqFfea0dXdd9vqai=hGuQ8kuc9pgc9s8qqaq=dirpe0xb9q8qiLsFr0=vr0=vr0dc8meaabaqaciaacaGaaeqabaqabeGadaaakeaacuWG4baEgaacamaaBaaaleaacqaIXaqmaeqaaOGaeiilaWIaeS47IWKaeiilaWIafmiEaGNbaGaadaWgaaWcbaGaemOBa4gabeaaaaa@3621@), (y˜1,⋯,y˜n
 MathType@MTEF@5@5@+=feaafiart1ev1aaatCvAUfKttLearuWrP9MDH5MBPbIqV92AaeXatLxBI9gBaebbnrfifHhDYfgasaacH8akY=wiFfYdH8Gipec8Eeeu0xXdbba9frFj0=OqFfea0dXdd9vqai=hGuQ8kuc9pgc9s8qqaq=dirpe0xb9q8qiLsFr0=vr0=vr0dc8meaabaqaciaacaGaaeqabaqabeGadaaakeaacuWG5bqEgaacamaaBaaaleaacqaIXaqmaeqaaOGaeiilaWIaeS47IWKaeiilaWIafmyEaKNbaGaadaWgaaWcbaGaemOBa4gabeaaaaa@3625@) where x˜i
 MathType@MTEF@5@5@+=feaafiart1ev1aaatCvAUfKttLearuWrP9MDH5MBPbIqV92AaeXatLxBI9gBaebbnrfifHhDYfgasaacH8akY=wiFfYdH8Gipec8Eeeu0xXdbba9frFj0=OqFfea0dXdd9vqai=hGuQ8kuc9pgc9s8qqaq=dirpe0xb9q8qiLsFr0=vr0=vr0dc8meaabaqaciaacaGaaeqabaqabeGadaaakeaacuWG4baEgaacamaaBaaaleaacqWGPbqAaeqaaaaa@2FBB@ = log_2 _(*x_i_*/*m*(*x*)), y˜i
 MathType@MTEF@5@5@+=feaafiart1ev1aaatCvAUfKttLearuWrP9MDH5MBPbIqV92AaeXatLxBI9gBaebbnrfifHhDYfgasaacH8akY=wiFfYdH8Gipec8Eeeu0xXdbba9frFj0=OqFfea0dXdd9vqai=hGuQ8kuc9pgc9s8qqaq=dirpe0xb9q8qiLsFr0=vr0=vr0dc8meaabaqaciaacaGaaeqabaqabeGadaaakeaacuWG5bqEgaacamaaBaaaleaacqWGPbqAaeqaaaaa@2FBD@ = log_2 _(*y_i_*/*m*(*y*)). The distance is zero when two expression profiles have identical fold-changes between all samples. When two profiles are close by this new distance measure, they also have high Pearson's correlation: when two profiles have zero distance by the new measure, the Pearson's correlation is one. When two profiles have high correlation, however, they are not necessarily close by the new distance measure. This property enables us to further select co-expressed genes among highly correlated genes. Figure 1 compares how two different expression distance measures correlate with the regulatory similarity in 7 data sets. The p-values and correlations are computed the way previously described for each data set and for each distance measure. Table 1 summarizes the result with seven different distance measures. All seven distance measures correlate with the regulatory distances. In contrast, when we use the Euclidian distance without any standardization, such correlations shown in Figure 1 disappear and the plots become noisy (not shown). This is because the probes in oligonucleotide arrays have different affinities; the signals from different probes are incomparable without a proper standardization. The figure and the table show that, while all other six distance measures perform similar, the new distance measure correlates best with the regulatory similarity. Only the new distance measure correlates significantly with the regulatory similarity in all seven data sets. Such improvement is expected since it is likely that many genes in the close regulatory distance at the molecular level should not only share their linear patterns but also have similar fold-changes across the samples. Interestingly, such link is extremely strong regardless of the choice of distance measure in the neurofibromatosis data, the only tumor data in our analysis. We attribute this to the inclusion of the transcription factors that are playing major role in this illness in the 147 available TFBMs in the binding analysis. Two main transcription factor complexes, NF-1 and NF-2, relating to the illness are included in the binding analysis.

**Table 1:** Correlations between median expression distance and regulatory similarity. The performance of different distance measures were compared in each of seven mouse experiments: Su et al. (Su), Storch et al. (Circadian), the neocortex development (Cortex), the murine model of human asthma (Lung), the hippocampus samples from neurofibromin-1 heterozygous study (NF), Zhao et al. (Muscle), and Wang et al.(PI). The number of microarrays used in each data set are shown in the first row. The p-values in the parentheses are obtained by permuting the mapping between genes and their promoter regions 500 times.

Expression distance measure	Su 89	Circadian 24	Cortex 17	Lung 39	NF 30	Muscle 54	PI 35
1 – correlation	-0.165 (0.422)	-0.261 (0.158)	-0.636 (0.008)	-0.423 (0.124)	-0.826 (0.000)	-0.358 (0.170)	-0.384 (0.198)
1 – cosine correlation	-0.802 (0.000)	-0.392 (0.106)	-0.679 (0.002)	-0.456 (0.066)	-0.878 (0.000)	-0.047 (0.488)	0.016 (0.666)
Square root 1 – correlation	-0.177 (0.416)	-0.280 (0.230)	-0.636 (0.008)	-0.412 (0.162)	-0.836 (0.000)	-0.401 (0.148)	-0.396 (0.200)
Square root 1 – cosine correlation	-0.783 (0.000)	-0.401 (0.166)	-0.683 (0.002)	-0.464 (0.064)	-0.869 (0.000)	-0.104 (0.490)	-0.007 (0.670)
1 – correlation after log2 transformation	-0.178 (0.310)	-0.254 (0.124)	-0.459 (0.032)	-0.534 (0.030)	-0.798 (0.000)	-0.136 (0.314)	-0.035 (0.428)
1 – cosine correlation after log2 transformation	-0.685 (0.006)	-0.026 (0.346)	-0.833 (0.000)	-0.830 (0.000)	-0.874 (0.000)	-0.540 (0.032)	0.346 (0.812)
The new distance measure	-0.777 (0.000)	-0.789 (0.000)	-0.735 (0.002)	-0.776 (0.000)	-0.859 (0.000)	-0.774 (0.000)	-0.656 (0.000)

## Discussion

We established that a simple expression distance measure that considers both the linear trends and expression fold-changes across the samples performs better than widely accepted distance measures do. Before clustering the gene expression profiles generated from oligonucleotide arrays, a proper standardization is essential because of the different affinities of the probes. We proposed a simple standardization that leads to the clustering of co-regulated genes more successfully than other widely used methods do. In addition, we demonstrated the correlation between the expression distance and the regulatory distance, in mouse. In yeast genome, the hypothesis that genes with similar expression patterns are likely to be regulated via the same mechanisms has been quantitatively tested with large-scale data [22]. Our study of such relationship between co-expression and co-regulation in a mammalian genome provide a groundwork for current efforts to develop the combined analysis methods for expression and cis-regulatory data. We note that, however, the statistically significant correlations are between regulatory similarity and median expression distance. The direct correlation between regulatory similarity and expression distance is not significant. This is expected because of our simplistic definition of regulatory similarity and the limited number (147) of known TFBMs from the murine genome. Here, we used the strength of such link to compare different expression distance measures but not to emphasize the correlation between the expression distance measure and the regulatory similarity. Following the work on yeast [22], we attempted to introduce the 2^nd ^order of regulatory distances by accounting indirect regulatory relationship between genes but the results were similar.

### Conceptual comparison of the new distance measure with others

**Figure 2:**
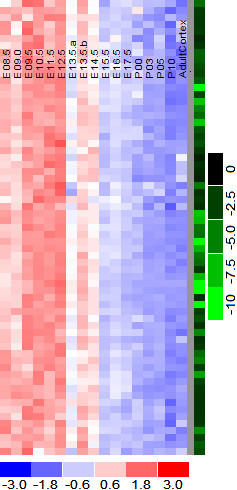
**Typical co-expressed gene cluster with high correlation. **The tightest gene cluster on the mice cortex developmental data is shown as a heatmap diagram; a sophisticated clustering algorithm is used with one minus correlation as the distance measure. The cluster consists 65 down regulated genes. The green column on the right side of the diagram shows the fold-change between two cortex samples at embryonic 8 days and adult age. The expression level matrix is standardized: mean subtracted and standard deviation divided; the color scheme ranges from -3 (blue, below the mean) to 3 (red, above the mean). The white color represents mean (0 value). The rows correspond to different genes, and the columns represent the experimental samples. The genes have tight linear expression pattern but their fold-changes between samples are highly variable. Such variability is a general phenomenon when one minus correlation is the distance measure.

One minus correlation is widely accepted as the distance measure between expression profiles; it captures the linear relationship between expression patterns. It fails, however, to account the fold-changes in expression between samples. When one minus correlation is the distance measure to cluster co-expressed gene groups, each cluster consist genes with similar linear expression patterns but with varying fold-changes between samples. As an illustration, Figure 2 shows a typical gene cluster in a heatmap diagram. It is the tightest gene cluster on the mice cortex developmental data generated by a sophisticated clustering algorithm [23]. The genes in the diagram have tight linear expression pattern but their fold-changes between samples are highly variable. Such variability is a general phenomenon when one minus correlation is the distance measure. This was the motivation to define a better distance measure; we hypothesized that many genes in the close regulatory distance at molecular level not only share their linear patterns but also have similar fold-changes in expression across the samples. In our experience, clustering analysis with correlation as the distance measure often results in large gene groups. In practice, it is desirable to reduce the gene numbers and increase the regulatory relevance since the genes are often the starting points for costly biological experiments. With the new distance measure, we identified gene clusters in mice cortex data with both similar linear pattern and similar fold-changes across the samples with smaller cluster sizes.

Another popular standardization approach is the Pearson's correlation on the log-transformed data. One minus Pearson's correlation is square root of Euclidian distance after centering and re-scaling the data. Hence, the regulatory information in the scale of log fold-changes is lost. In addition, no longer the genes with similar linear trends will cluster together. In contrast, the new distance measure, after log transformation, involves centering but not re-scaling. Table 1 suggests that the scale of log fold-changes contains significant co-regulation information.

The preservation of the inherent regulatory information in expression profiles depend on both the hybridization process and the expression index calculation methods. Here, all samples were hybridized according to the Affymetrix protocol and the expression indices were computed by the multi-array model based approach [24].

### Meta data analysis

We proposed the log transformation with base 2. When expression data from multiple experiments are combined, however, often variation from certain experiments dominates the analysis. We suggest using different bases for log-transformation in each experiment, e.g., 2^80th percentile of the interquantile ranges of all genes^.

## Conclusion

The study reveals that the proposed distance measure for comparing expression profiles reflects the inherent regulatory information better than widely accepted distance measures such as the Pearson's correlation or cosine correlation, with or without log transformation. The distance measure enables us to identify genes with large number of common regulatory elements.

## Methods

### Microarray data

For each data set, the DNA-Chip Analyzer (dChip) was used to normalize all CEL files to the baseline array and compute the PM/MM model-based expression [25]. For each data set, 1000 probe sets with the largest coefficient of variation and with presence call percentage larger than 20% underwent the subsequent analyses. These probe sets were filtered to have non-redundant Affymetrix probe set ID's and non-redundant NCBI RefSeq ID's.

### Binding data

We collected 147 position weight matrices (PWM) for mouse transcription factor binding sites from TRANSFAC. The PWMs were mapped to the promoter regions (from 5 kb upstream to 1 kb downstream relative to the transcriptional start site) of all genes (12079 non redundant RefSeq IDs) in the two mouse Affymetrix mRNA chips, MG_U74av2 and MOE430a. For each PWM and each gene, a sliding window was used to scan the promoter sequence and a likelihood ratio *R *between the motif model and the background model was computed for each window. The motif was modelled by a Product Dirichlet distribution whose parameters were defined by PWM. The background was modelled by a third order Markov chain, and the transition probability matrix was estimated from all genes' sequences. For each window, a motif score *S *was computed as *S *= -∑_*i *_log(*θ_ix_*)*I*{*b_i _*= *x*}, where *b*_*i *_is the *i*^th ^base of the window, *x *∊ {*A,C,G,T*} and *θ*_*ix *_represents the probability of observing *x *in the *i*^th ^position of the motif and was derived from PWM. A window was called as a binding site if its likelihood ratio *R*>100 and the observed motif score, *S*_*obs*_, satisfies Pr(*S*<*S*_*obs *_| PWM) >0.05. The selected binding sites were then filtered by the cross-species alignment score derived from human-mouse-rat whole genomes: only binding sites in the regions with the top 10% scores in the genome were preserved. *R *of all the preserved binding sites were then added up, and the sum was adjusted by a factor 6000/*L*_*c*_, where *L*_*c *_is the number of all bases, in the -5 kb~+1 kb promoter region, that have cross-species alignment score greater than top 10 % of the genome. This adjusted sum *M*_*R *_was transformed as log(*M*_*R *_+1) and used as the motif mapping score for that specific gene and PWM.

To compute the cross-species alignment score, MULTIZ alignment of human, mouse and rat was downloaded from UCSC. A 50 base pair sliding window was used to scan the alignment. A z-score defined by zhm=(pobs−p)/p(1−p)/n
 MathType@MTEF@5@5@+=feaafiart1ev1aaatCvAUfKttLearuWrP9MDH5MBPbIqV92AaeXatLxBI9gBaebbnrfifHhDYfgasaacH8akY=wiFfYdH8Gipec8Eeeu0xXdbba9frFj0=OqFfea0dXdd9vqai=hGuQ8kuc9pgc9s8qqaq=dirpe0xb9q8qiLsFr0=vr0=vr0dc8meaabaqaciaacaGaaeqabaqabeGadaaakeaacqWG6bGEdaWgaaWcbaGaemiAaGMaemyBa0gabeaakiabg2da9maalyaabaGaeiikaGIaemiCaa3aaSbaaSqaaiabd+gaVjabdkgaIjabdohaZbqabaGccqGHsislcqWGWbaCcqGGPaqkaeaadaGcaaqaamaalyaabaGaemiCaaNaeiikaGIaeGymaeJaeyOeI0IaemiCaaNaeiykaKcabaGaemOBa4gaaaWcbeaaaaaaaa@43F8@ was computed for each window. *p*_*obs *_is the percent identity of human-mouse alignment in the window; *n *is the number of columns in human-mouse alignment that are not gap vs. gap (i.e. the denominator used to derive *p*_*obs*_); *p *is the percent identity of human-mouse alignments in the surrounding 1 Mb window, and it controls for the regional variation of dissimilarity. A similar z-score, *z*_*hr*_, was computed for human-rat alignment, and the mean of *z*_*hm *_and *z*_*hr *_was used as the final score for the window. The cross-species alignment score for each base was then defined as the maximum score of all the windows that covers the base.

**Figure 3:**
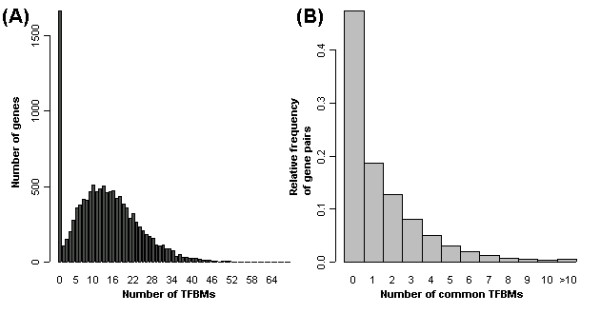
**Overview of the binding data. **(a) The histogram of the number of the known TFBMs in the promoter region of 12,079 non-redundant genes. (b) The distribution of the number of common known TFBSs in the promoter regions of all 72,945,081 gene pairs in 2 mouse chips.

Then for each PWM, we treat it to be present in the promoter of a gene when the mapping score *M*_*R *_is in the top 10% of same PWM's scores. Figure 3a shows the histogram of the number of the known TFBMs in the promoter region of each of 12,079 non-redundant genes. Figure 3b is the relative frequency of the number of common known TFBMs in the promoter regions of all 72,945,081 gene pairs in 2 mouse chips.

### Regulatory similarity

We define regulatory similarity between two genes as the number of common known TFBMs on their promoter regions. Although the binding of transcription factor is not always equivalent to the regulation by the transcription factor, the shared transcription factor binding is a good approximation for co-regulation [22].

### Metrics for comparing expression profiles

Seven distance measures for comparing expression profiles were considered: 1 minus correlation, 1 minus cosine correlation, square root of 1 minus correlation, square root of 1 minus cosine correlation, 1 minus correlation after log2 transformation, 1 minus cosine correlation after log2 transformation, and our proposed distance measure (See table 2).

**Table 2:** Distance measures between two expression profiles. Two expression profiles *x *= (*x*_1_, ..., *x*_n_, *y *= (*y*_1_, ..., *y*_n_) have medians *m*(*x*), *m*(*y*) and the means x¯
 MathType@MTEF@5@5@+=feaafiart1ev1aaatCvAUfKttLearuWrP9MDH5MBPbIqV92AaeXatLxBI9gBaebbnrfifHhDYfgasaacH8akY=wiFfYdH8Gipec8Eeeu0xXdbba9frFj0=OqFfea0dXdd9vqai=hGuQ8kuc9pgc9s8qqaq=dirpe0xb9q8qiLsFr0=vr0=vr0dc8meaabaqaciaacaGaaeqabaqabeGadaaakeaacuWG4baEgaqeaaaa@2E3D@, y¯
 MathType@MTEF@5@5@+=feaafiart1ev1aaatCvAUfKttLearuWrP9MDH5MBPbIqV92AaeXatLxBI9gBaebbnrfifHhDYfgasaacH8akY=wiFfYdH8Gipec8Eeeu0xXdbba9frFj0=OqFfea0dXdd9vqai=hGuQ8kuc9pgc9s8qqaq=dirpe0xb9q8qiLsFr0=vr0=vr0dc8meaabaqaciaacaGaaeqabaqabeGadaaakeaacuWG5bqEgaqeaaaa@2E3F@. The arithmetic relationship between the measures is as following: E(x,y)=2(n−1)(1−rx,y)
 MathType@MTEF@5@5@+=feaafiart1ev1aaatCvAUfKttLearuWrP9MDH5MBPbIqV92AaeXatLxBI9gBaebbnrfifHhDYfgasaacH8akY=wiFfYdH8Gipec8Eeeu0xXdbba9frFj0=OqFfea0dXdd9vqai=hGuQ8kuc9pgc9s8qqaq=dirpe0xb9q8qiLsFr0=vr0=vr0dc8meaabaqaciaacaGaaeqabaqabeGadaaakeaacqWGfbqrcqGGOaakcuWG4baEgaaeaiabcYcaSiqbdMha5zaaqaGaeiykaKIaeyypa0ZaaOaaaeaacqaIYaGmcqGGOaakcqWGUbGBcqGHsislcqaIXaqmcqGGPaqkcqGGOaakcqaIXaqmcqGHsislcqWGYbGCdaWgaaWcbaGaemiEaGNaeiilaWIaemyEaKhabeaakiabcMcaPaWcbeaaaaa@4380@, E(x˜,y˜)=2n(1−rx,ycos⁡)
 MathType@MTEF@5@5@+=feaafiart1ev1aaatCvAUfKttLearuWrP9MDH5MBPbIqV92AaeXatLxBI9gBaebbnrfifHhDYfgasaacH8akY=wiFfYdH8Gipec8Eeeu0xXdbba9frFj0=OqFfea0dXdd9vqai=hGuQ8kuc9pgc9s8qqaq=dirpe0xb9q8qiLsFr0=vr0=vr0dc8meaabaqaciaacaGaaeqabaqabeGadaaakeaacqWGfbqrcqGGOaakcuWG4baEgaacaiabcYcaSiqbdMha5zaaiaGaeiykaKIaeyypa0ZaaOaaaeaacqaIYaGmcqWGUbGBcqGGOaakcqaIXaqmcqGHsislcqWGYbGCdaqhaaWcbaGaemiEaGNaeiilaWIaemyEaKhabaGagi4yamMaei4Ba8Maei4CamhaaOGaeiykaKcaleqaaaaa@4406@ and *E*(*x'*, *y'*) = *d*(*x*, *y*) where  xi=(xi−x¯)/∑(xi−x¯)2/(n−1)
 MathType@MTEF@5@5@+=feaafiart1ev1aaatCvAUfKttLearuWrP9MDH5MBPbIqV92AaeXatLxBI9gBaebbnrfifHhDYfgasaacH8akY=wiFfYdH8Gipec8Eeeu0xXdbba9frFj0=OqFfea0dXdd9vqai=hGuQ8kuc9pgc9s8qqaq=dirpe0xb9q8qiLsFr0=vr0=vr0dc8meaabaqaciaacaGaaeqabaqabeGadaaakeaacqqGGaaicuWG4baEgaaeamaaBaaaleaacqWGPbqAaeqaaOGaeyypa0ZaaSGbaeaacqGGOaakcqWG4baEdaWgaaWcbaGaemyAaKgabeaakiabgkHiTiqbdIha4zaaraGaeiykaKcabaWaaOaaaeaadaWcgaqaamaaqaeabaGaeiikaGIaemiEaG3aaSbaaSqaaiabdMgaPbqabaGccqGHsislcuWG4baEgaqeaiabcMcaPmaaCaaaleqabaGaeGOmaidaaaqabeqaniabggHiLdaakeaacqGGOaakcqWGUbGBcqGHsislcqaIXaqmcqGGPaqkaaaaleqaaaaaaaa@487B@, x˜i=xi/∑xi2/n
 MathType@MTEF@5@5@+=feaafiart1ev1aaatCvAUfKttLearuWrP9MDH5MBPbIqV92AaeXatLxBI9gBaebbnrfifHhDYfgasaacH8akY=wiFfYdH8Gipec8Eeeu0xXdbba9frFj0=OqFfea0dXdd9vqai=hGuQ8kuc9pgc9s8qqaq=dirpe0xb9q8qiLsFr0=vr0=vr0dc8meaabaqaciaacaGaaeqabaqabeGadaaakeaacuWG4baEgaacamaaBaaaleaacqWGPbqAaeqaaOGaeyypa0ZaaSGbaeaacqWG4baEdaWgaaWcbaGaemyAaKgabeaaaOqaamaakaaabaWaaSGbaeaadaaeabqaaiabdIha4naaDaaaleaacqWGPbqAaeaacqaIYaGmaaaabeqab0GaeyyeIuoaaOqaaiabd6gaUbaaaSqabaaaaaaa@3B87@, and x′i
 MathType@MTEF@5@5@+=feaafiart1ev1aaatCvAUfKttLearuWrP9MDH5MBPbIqV92AaeXatLxBI9gBaebbnrfifHhDYfgasaacH8akY=wiFfYdH8Gipec8Eeeu0xXdbba9frFj0=OqFfea0dXdd9vqai=hGuQ8kuc9pgc9s8qqaq=dirpe0xb9q8qiLsFr0=vr0=vr0dc8meaabaqaciaacaGaaeqabaqabeGadaaakeaacuWG4baEgaqbamaaBaaaleaacqWGPbqAaeqaaaaa@2FB8@ = log_2 _(*x*/*m*(*x*)) and  yi
 MathType@MTEF@5@5@+=feaafiart1ev1aaatCvAUfKttLearuWrP9MDH5MBPbIqV92AaeXatLxBI9gBaebbnrfifHhDYfgasaacH8akY=wiFfYdH8Gipec8Eeeu0xXdbba9frFj0=OqFfea0dXdd9vqai=hGuQ8kuc9pgc9s8qqaq=dirpe0xb9q8qiLsFr0=vr0=vr0dc8meaabaqaciaacaGaaeqabaqabeGadaaakeaacqqGGaaicuWG5bqEgaaeamaaBaaaleaacqWGPbqAaeqaaaaa@308C@, y˜i
 MathType@MTEF@5@5@+=feaafiart1ev1aaatCvAUfKttLearuWrP9MDH5MBPbIqV92AaeXatLxBI9gBaebbnrfifHhDYfgasaacH8akY=wiFfYdH8Gipec8Eeeu0xXdbba9frFj0=OqFfea0dXdd9vqai=hGuQ8kuc9pgc9s8qqaq=dirpe0xb9q8qiLsFr0=vr0=vr0dc8meaabaqaciaacaGaaeqabaqabeGadaaakeaacuWG5bqEgaacamaaBaaaleaacqWGPbqAaeqaaaaa@2FBD@, y′i
 MathType@MTEF@5@5@+=feaafiart1ev1aaatCvAUfKttLearuWrP9MDH5MBPbIqV92AaeXatLxBI9gBaebbnrfifHhDYfgasaacH8akY=wiFfYdH8Gipec8Eeeu0xXdbba9frFj0=OqFfea0dXdd9vqai=hGuQ8kuc9pgc9s8qqaq=dirpe0xb9q8qiLsFr0=vr0=vr0dc8meaabaqaciaacaGaaeqabaqabeGadaaakeaacuWG5bqEgaqbamaaBaaaleaacqWGPbqAaeqaaaaa@2FBA@ defined similarly.

Distance Measures	Definition
Correlation	rx,y=∑1n(xi−x¯)(yi−y¯)/∑1n(xi−x¯)2∑1n(yi−y¯)2 MathType@MTEF@5@5@+=feaafiart1ev1aaatCvAUfKttLearuWrP9MDH5MBPbIqV92AaeXatLxBI9gBaebbnrfifHhDYfgasaacH8akY=wiFfYdH8Gipec8Eeeu0xXdbba9frFj0=OqFfea0dXdd9vqai=hGuQ8kuc9pgc9s8qqaq=dirpe0xb9q8qiLsFr0=vr0=vr0dc8meaabaqaciaacaGaaeqabaqabeGadaaakeaacqWGYbGCdaWgaaWcbaGaemiEaGNaeiilaWIaemyEaKhabeaakiabg2da9maalyaabaWaaabCaeaacqGGOaakcqWG4baEdaWgaaWcbaGaemyAaKgabeaakiabgkHiTiqbdIha4zaaraGaeiykaKIaeiikaGIaemyEaK3aaSbaaSqaaiabdMgaPbqabaGccqGHsislcuWG5bqEgaqeaiabcMcaPaWcbaGaeGymaedabaGaemOBa4ganiabggHiLdaakeaadaGcaaqaamaaqahabaGaeiikaGIaemiEaG3aaSbaaSqaaiabdMgaPbqabaGccqGHsislcuWG4baEgaqeaiabcMcaPmaaCaaaleqabaGaeGOmaidaaaqaaiabigdaXaqaaiabd6gaUbqdcqGHris5aOWaaabCaeaacqGGOaakcqWG5bqEdaWgaaWcbaGaemyAaKgabeaakiabgkHiTiqbdMha5zaaraGaeiykaKYaaWbaaSqabeaacqaIYaGmaaaabaGaeGymaedabaGaemOBa4ganiabggHiLdaaleqaaaaaaaa@604B@
Cosine correlation	rx,ycos⁡=∑1nxiyi/∑1nxi2∑1nyi2 MathType@MTEF@5@5@+=feaafiart1ev1aaatCvAUfKttLearuWrP9MDH5MBPbIqV92AaeXatLxBI9gBaebbnrfifHhDYfgasaacH8akY=wiFfYdH8Gipec8Eeeu0xXdbba9frFj0=OqFfea0dXdd9vqai=hGuQ8kuc9pgc9s8qqaq=dirpe0xb9q8qiLsFr0=vr0=vr0dc8meaabaqaciaacaGaaeqabaqabeGadaaakeaacqWGYbGCdaqhaaWcbaGaemiEaGNaeiilaWIaemyEaKhabaGagi4yamMaei4Ba8Maei4CamhaaOGaeyypa0ZaaSGbaeaadaaeWbqaaiabdIha4naaBaaaleaacqWGPbqAaeqaaOGaemyEaK3aaSbaaSqaaiabdMgaPbqabaaabaGaeGymaedabaGaemOBa4ganiabggHiLdaakeaadaGcaaqaamaaqahabaGaemiEaG3aa0baaSqaaiabdMgaPbqaaiabikdaYaaaaeaacqaIXaqmaeaacqWGUbGBa0GaeyyeIuoakmaaqahabaGaemyEaK3aa0baaSqaaiabdMgaPbqaaiabikdaYaaaaeaacqaIXaqmaeaacqWGUbGBa0GaeyyeIuoaaSqabaaaaaaa@532B@
Euclidian distance	E(x,y)=∑1n(xi−yi)2 MathType@MTEF@5@5@+=feaafiart1ev1aaatCvAUfKttLearuWrP9MDH5MBPbIqV92AaeXatLxBI9gBaebbnrfifHhDYfgasaacH8akY=wiFfYdH8Gipec8Eeeu0xXdbba9frFj0=OqFfea0dXdd9vqai=hGuQ8kuc9pgc9s8qqaq=dirpe0xb9q8qiLsFr0=vr0=vr0dc8meaabaqaciaacaGaaeqabaqabeGadaaakeaacqWGfbqrcqGGOaakcqWG4baEcqGGSaalcqWG5bqEcqGGPaqkcqGH9aqpdaGcaaqaamaaqahabaGaeiikaGIaemiEaG3aaSbaaSqaaiabdMgaPbqabaGccqGHsislcqWG5bqEdaWgaaWcbaGaemyAaKgabeaakiabcMcaPmaaCaaaleqabaGaeGOmaidaaaqaaiabigdaXaqaaiabd6gaUbqdcqGHris5aaWcbeaaaaa@42C6@
New distance measure	d(x,y)=∑i=1n{log⁡2(xi/m(x))−log⁡2(yi/m(y))}2 MathType@MTEF@5@5@+=feaafiart1ev1aaatCvAUfKttLearuWrP9MDH5MBPbIqV92AaeXatLxBI9gBaebbnrfifHhDYfgasaacH8akY=wiFfYdH8Gipec8Eeeu0xXdbba9frFj0=OqFfea0dXdd9vqai=hGuQ8kuc9pgc9s8qqaq=dirpe0xb9q8qiLsFr0=vr0=vr0dc8meaabaqaciaacaGaaeqabaqabeGadaaakeaacqWGKbazcqGGOaakcqWG4baEcqGGSaalcqWG5bqEcqGGPaqkcqGH9aqpdaGcaaqaamaaqahabaWaaiWaaeaacyGGSbaBcqGGVbWBcqGGNbWzdaWgaaWcbaGaeGOmaidabeaakiabcIcaOmaalyaabaGaemiEaG3aaSbaaSqaaiabdMgaPbqabaaakeaacqWGTbqBcqGGOaakcqWG4baEcqGGPaqkaaGaeiykaKIaeyOeI0IagiiBaWMaei4Ba8Maei4zaC2aaSbaaSqaaiabikdaYaqabaGccqGGOaakdaWcgaqaaiabdMha5naaBaaaleaacqWGPbqAaeqaaaGcbaGaemyBa0MaeiikaGIaemyEaKNaeiykaKcaaiabcMcaPaGaay5Eaiaaw2haamaaCaaaleqabaGaeGOmaidaaaqaaiabdMgaPjabg2da9iabigdaXaqaaiabd6gaUbqdcqGHris5aaWcbeaaaaa@5D1E@

### Significance of the correlation for comparing expression distance and regulatory similarity

The correlation between the median expression distance and the regulatory similarity is computed from all possible gene pairs. To calculate the significance of such correlation, for each data set, we permuted the mapping between genes and their promoter regions 500 times and computed correlation between the median expression distance and the regulatory similarity. The p-value is the number of correlations equal or below the observed correlation. Note that, as the regulatory similarity increases, the standard deviation of median expression distance becomes large because the number of gene pairs decrease. When fewer than 5 gene pairs have certain regulatory similarity, the median expression distance is computed after combining the nearest regulatory similarities to make each point in the plots represent at least 5 gene pairs.

## Authors' contributions

RSK conceived of the study, collected the public expression data, performed statistical analysis, and drafted the manuscript. HJ carried out the binding analysis and helped to draft the manuscript. WHW advised the study and helped to draft the manuscript. All authors read and approved the final manuscript.
